# Insights into the role of endonuclease V in RNA metabolism in *Trypanosoma brucei*

**DOI:** 10.1038/s41598-017-08910-1

**Published:** 2017-08-17

**Authors:** Daniel García-Caballero, Guiomar Pérez-Moreno, Antonio M. Estévez, Luis Miguel Ruíz-Pérez, Antonio E. Vidal, Dolores González-Pacanowska

**Affiliations:** Departmento de Bioquímica y Farmacología Molecular, Instituto de Parasitología y Biomedicina Lopez-Neyra, Consejo Superior de Investigaciones Científicas (CSIC), Armilla (Granada), 18016 Spain

## Abstract

Inosine may arise in DNA as a result of oxidative deamination of adenine or misincorporation of deoxyinosine triphosphate during replication. On the other hand, the occurrence of inosine in RNA is considered a normal and essential modification induced by specific adenosine deaminases acting on mRNA and tRNA. In prokaryotes, endonuclease V (EndoV) can recognize and cleave inosine-containing DNA. In contrast, mammalian EndoVs preferentially cleave inosine-containing RNA, suggesting a role in RNA metabolism for the eukaryotic members of this protein family. We have performed a biochemical characterization of EndoV from the protozoan parasite *Trypanosoma brucei*. *In vitro*, *Tb*EndoV efficiently processes single-stranded RNA oligonucleotides with inosine, including A to I-edited tRNA-like substrates but exhibits weak activity over DNA, except when a ribonucleotide is placed 3′ to the inosine. Immunolocalization studies performed in procyclic forms indicate that *Tb*EndoV is mainly cytosolic yet upon nutritional stress it redistributes and accumulates in stress granules colocalizing with the DEAD-box helicase *Tb*Dhh1. RNAi-mediated depletion of *Tb*EndoV results in moderate growth defects in procyclic cells while the two *EndoV* alleles could be readily knocked out in bloodstream forms. Taken together, these observations suggest an important role of *Tb*EndoV in RNA metabolism in procyclic forms of the parasite.

## Introduction

Nucleic acids are continuously exposed to endogenous and environmental damaging agents. Among the most common DNA lesions are nitrogenous base deaminations, which may become a major threat to genetic integrity. Deamination in DNA and RNA can also be enzyme-mediated in a controlled fashion and has an essential role in multiple biological processes^1–4^. Undesired deamination if not repaired, can give rise to mutations during semiconservative replicative synthesis, since loss of the amino group changes its pairing properties^5^. For instance, uracil (U), the product of cytosine deamination, pairs with adenine while deaminated adenine or hypoxanthine (Hx) is usually read as G by the DNA polymerase therefore inducing GC > AT and AT > GC transitions respectively. To avoid mutations, these non-canonical bases are mainly removed from DNA by specific DNA glycosylases through the base excision repair (BER) pathway^6^. Early works reported the purification from *E*. *coli* of an endodeoxyribonuclease (endonuclease V, EndoV) that also recognizes and nicks DNA containing deaminated bases and therefore could be part of an alternative excision repair (AER) pathway for the elimination of U and Hx from DNA^7, 8^. Besides deaminated bases, EndoV can also cleave an extensive array of damaged DNA lesions including AP sites, mismatched base pairs and flap and pseudo-Y DNA structures^8–14^. In spite of its broad substrate specificity, biochemical and genetic evidences led to conclude a preferential role in the repair of deoxyinosine (the deoxynucleoside containing Hx) in double- and single-stranded DNA^8, 15, 16^. In both cases, EndoV catalyzes the cleavage of the second phosphodiester bond 3′ to the lesion without releasing the erroneous nucleotide^14^. Hence, additional enzymatic factors are required to eliminate the lesion and complete the repair although they have not been yet identified. *In vitro*, purified DNA polymerase I and DNA ligase appear sufficient to reconstitute the repair process^17, 18^.

EndoV homologues are widely conserved throughout bacteria, archaea and eukaryotes but with differences in substrate specificity that likely imply some degree of divergence in their cellular functions. Although *E*. *coli* EndoV has been described as a DNA repair enzyme for many years, in fact, it can also act on inosine at RNA with equal efficiency^19^. Similarly, the enzyme of the *Pyrococcus furiosus* archaeon shows strong activity toward RNA substrates as well as DNA substrates^20^. In contrast, mouse and human EndoVs exhibit very weak activity over deoxyinosine and DNA substrates in general^21, 22^ whereas they incise inosine-containing RNA very efficiently, suggesting a role in RNA metabolism instead of DNA repair for the mammalian enzymes^19, 23, 24^.

*Trypanosoma brucei* is a protozoan parasite belonging to the Kinetoplastida order and the causative agent of African trypanosomiasis and nagana in humans and animals, respectively^25^. During its life cycle, *T*. *brucei* alternates between an insect and a mammalian host and the adaptation to the environmental conditions requires significant structural and physiological changes. During infection, an essential component of the primary immune response is the production of nitric oxide (NO) by activated phagocytes that generates reactive oxygen and nitrogen species (ROS and RNS). However, the impact of DNA repair mechanisms such as endonuclease V to counteract host-generated oxidative and nitrosative stress has not been yet investigated in these human pathogens and may be relevant to understand their capacity to survive in such adverse environment. Here, we have purified and characterized the catalytic properties of the endonuclease V encoded by *Trypanosoma brucei* genome (*Tb*EndoV). *In vitro*, T*b*EndoV efficiently processes inosine-containing single-stranded RNA oligonucleotides, including A to I-edited tRNA-like substrates. On the contrary, a weak activity was observed over inosine in DNA, except when a ribonucleotide was placed 3′ to the lesion. Analysis by immunofluorescence microscopy indicates that *Tb*EndoV localizes predominantly to the cytosol. The enzyme appears to be non-essential in bloodstream forms (mammalian-stage parasites) where the two *EndoV* alleles could be readily knocked out. In contrast, protein depletion in procyclic forms (insect-stage parasites) by RNA interference led to impaired growth and defects in cell cycle progression, thus suggesting a specific and vital role for *Tb*EndoV at this life stage.

## Results

### Identification of a putative endonuclease V in *Trypanosoma brucei*

**Figure 1:**
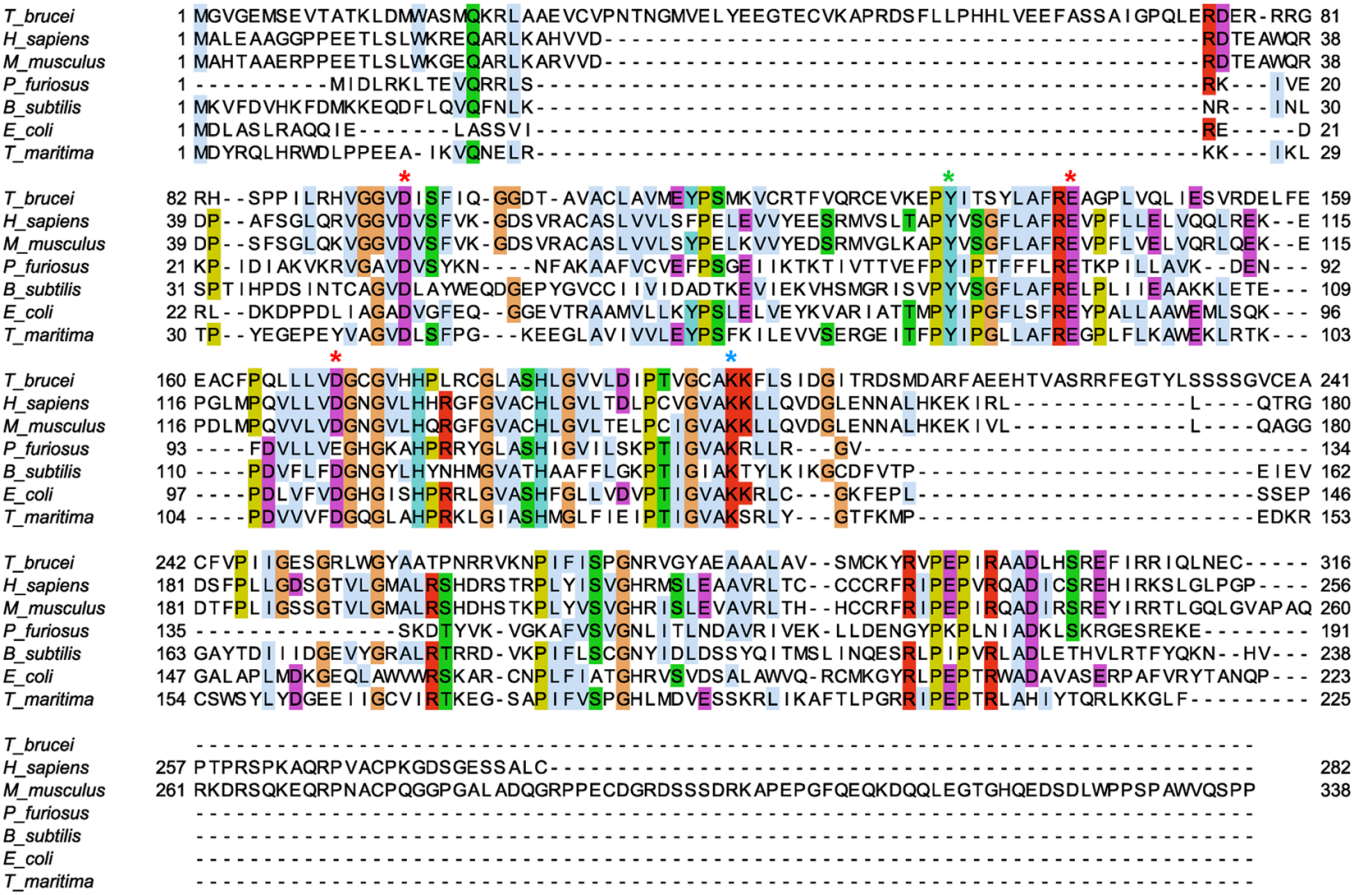
Sequence alignment of endonuclease V orthologs from several organisms including *T*. *brucei*. The alignment was made in ClustalOmega and visualized with JalView. Amino acids are colored using the clustal color scheme (for more details check the following web page: http://www.jalview.org/help/html/colourSchemes/clustal.html) and the identity threshold was set at 50%. The amino acids involved in metal cofactor interaction are indicated by red asterisks, the amino acids responsible for substrate-enzyme interaction are indicated by green asterisks and the amino acids involved in product-enzyme interaction are marked with blue asterisks. Protein sequences were retrieved from NCBI RefSeq: *T_brucei*, *Trypanosoma brucei* (XP_822927.1); *H_sapiens*, *Homo sapiens* (NP_775898.2); *M_musculus*, *Mus musculus* (NP_001158108.1); *P_furiosus*, *Pyrococcus furiosus* (AFN03782.1); *B_subtilis*, *Bacillus subtilis* (NP_391498.2); *E._coli*, *Escherichia coli* (NP_418426.2); *T_maritima*, *Thermotoga maritima* (NP_229661.1).

Homology searching of the *Trypanosoma brucei brucei* genome database, using the human endonuclease V ortholog as the query sequence, identified a protein annotated as a putative endonuclease V (Tb927.10.6860) composed of 316 amino acids and a calculated mass of 34.9 kDa. The *T*. *brucei* EndoV protein sequence was aligned with other six characterized orthologs from prokaryotic and eukaryotic organisms (Fig. 1). Three amino acids identified as crucial for endonuclease activity in *Thermotoga maritima*, Asp43, Glu89 and Asp110, are conserved in *Tb*EndoV. These residues coordinate the interaction with the metal cofactor which is essential for catalysis^26^. Sequence comparison revealed that, *Tb*EndoV exhibits a high similarity to the human ortholog (identity = 37%; E = 3 × 10^−41^; bits = 143).

### *T*. *brucei* endonuclease V preferentially cleaves inosine-containing RNA

To characterize the enzymatic properties of *Tb*EndoV, the corresponding open reading frame was amplified by PCR and cloned into an *E*. *coli* vector that allows expression of the protein fused to the maltose-binding protein in order to increase its expression and solubility. The MBP-*Tb*EndoV protein was overexpressed in the *E*. *coli* TB1 strain and purified to over 99% purity by amylose affinity and gel filtration chromatography.

**Figure 2:**
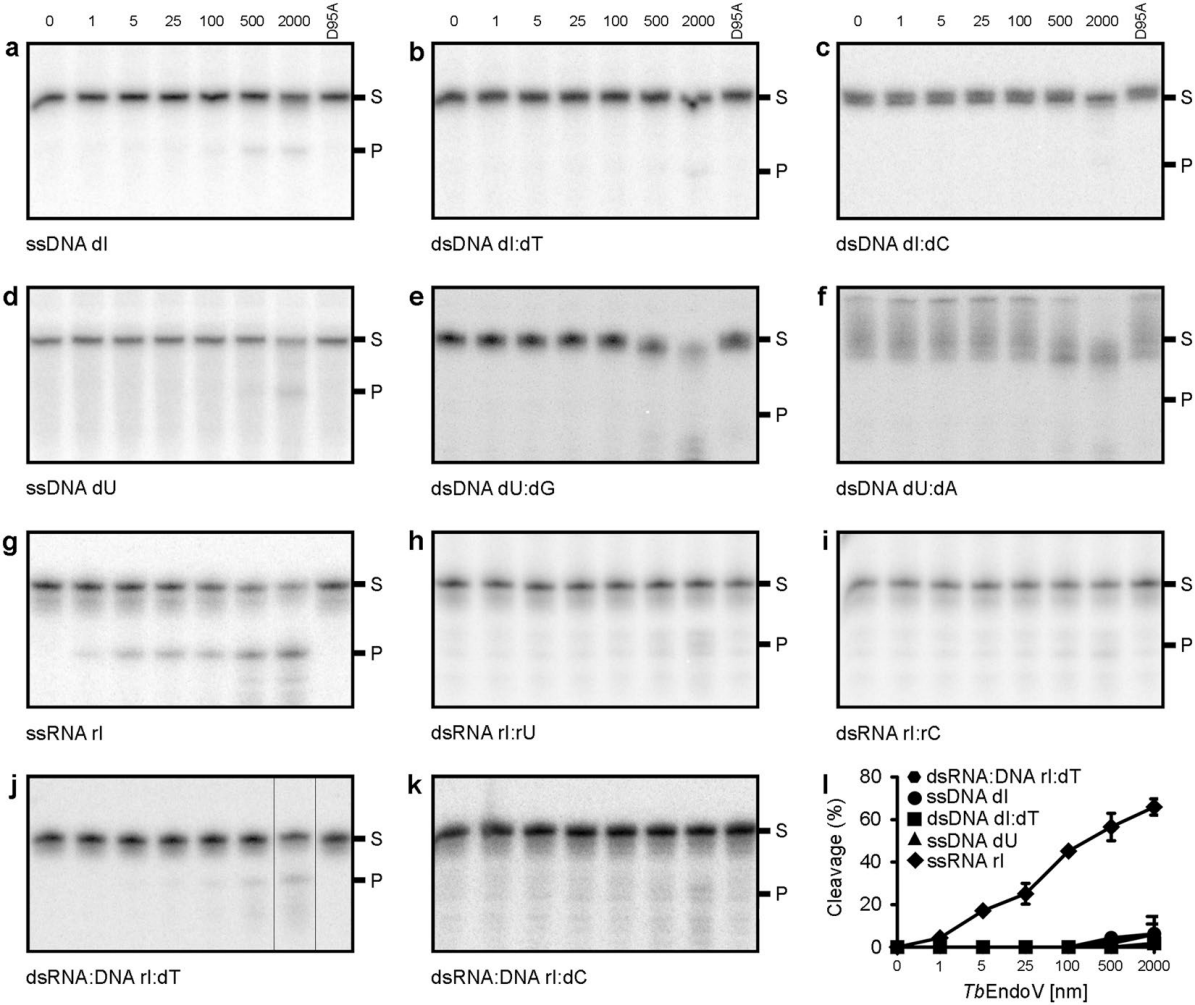
*Tb*EndoV acts efficiently upon inosine-containing ssRNA. DNA substrates with (**a**–**c**) inosine or (**d**–**f**) uracil, and (**g**) single and (**h**,**i**) double-strand RNA substrates or (**j,k**) RNA-DNA hybrids were incubated with increasing amounts of *Tb*EndoV (0–2000 nM, as indicated). (**l**) Plot of the percentage of substrate cleaved by endonuclease V activity versus the total amount of protein used in the reaction. Each point represents the mean (±s.d.) from three independent experiments. The D95A catalytic mutant was used as negative control. Reaction products were analyzed by 20% denaturing PAGE. Image in j was cropped and edited for easier comparison. Positions of the reaction substrate (S) and product (P) are indicated.

The purified recombinant protein was initially assayed for endonuclease activity on 5′-radiolabeled 21-nucleotide single- and double-strand DNA substrates in which one strand contained hypoxanthine or uracil in a central position. Oligonucleotide sequences can be found in Fig. S1. Double-strand substrates were designed to produce Hx:T (dsDNA dI:dT), Hx:C (dsDNA dI:dC), U:G (dsDNA dU:dG) and U:A (dsDNA dU:dA) base pairs that mimic potentially physiological DNA substrates. The dsDNA dI:dT and dsDNA dU:dG substrates may result from adenine and cytosine deamination at A:T and C:G base pairs respectively while dsDNA dI:dC and dsDNA dU:dA can be generated during replicative synthesis of hypoxanthine- or uracil-containing DNA strands, respectively. In general, *Tb*EndoV showed poor or no endonuclease activity on these DNA substrates (Fig. 2a–f). Indeed, ssDNA dI and dsDNA dI:dT were only cleaved at very high enzyme concentrations while practically no cleavage was observed with dsDNA dI:dC (Fig. 2a–c). The trypanosomal enzyme also exhibits residual activity on uracil in ssDNA substrates while no activity was detected over the uracil-containing dsDNA substrates (Fig. 2d–f). To rule out a possible contamination with bacterial endonuclease V during purification, we generated a D95A catalytic mutant by site-directed mutagenesis. The aspartate at this position is a highly conserved residue responsible for the coordination of the metal cofactor during substrate cleavage^27^. As shown in Fig. 2, replacement of aspartate 95 by alanine fully abolished *Tb*EndoV activity in agreement with studies on homologous enzymes^19, 28, 29^. Hence, the activity detected is intrinsic to *Tb*EndoV.

While bacterial EndoV cleaves both DNA and RNA substrates, the human enzyme has been reported to act more efficiently upon inosine-containing RNA substrates^19, 23^. Indeed, riboinosine can arise by spontaneous or chemically-induced adenine deamination, as a consequence of enzymatic RNA-editing or by erroneous incorporation of ITP during transcription^30^. We therefore tested the activity of *Tb*EndoV using different RNA substrates (Fig. 2). As shown in Fig. 2g, trypanosomal EndoV efficiently cleaves single-strand RNA substrates with inosine (ssRNA rI) but is not able to process double-strand RNA when Hx pairs with U (dsRNA rI:rU) or cytosine (dsRNA rI:rC) (Fig. 2h,i). As expected, the incubation of the RNA substrates with the D95A catalytic mutant did not result in product formation.

We have also considered the possibility that riboinosine could be incorporated into nascent RNA during transcription giving rise to a transient RNA:DNA hybrid where Hx would establish a stable base pair with C. However, the cleavage assay did not reveal significant endonuclease activity using the dsRNA:DNA rI:dC substrate and only minor incision was detected with a similar substrate containing an Hx:T mispair (dsRNA:DNA rI:dT) (Fig. 2j,k).

Once ssRNA rI was established as the preferred substrate for *Tb*EndoV (Fig. 2l), reaction conditions were empirically varied to determine how they affected its incision activity (Fig. S2). We found that *Tb*EndoV acts more efficiently at pH 7.5 when using 1 mM MnCl_2_, the preferred metal cofactor (Fig. S2a,c). On the other hand, addition of high concentrations of NaCl or KCl (≥100 mM) greatly inhibited *Tb*EndoV activity and therefore, salts were not included in the final reaction buffer (Fig. S2b). We also noticed that the ssRNA rI substrate was refractory to undergo complete cleavage by *Tb*EndoV even at high enzyme concentrations and under different reaction conditions tested. In contrast, total incision was achieved with an alternative substrate sequence, indicating that substrate recognition and/or incision is sequence-dependent and activity could be influenced by the secondary structure of the substrate (Fig. S2d).

### Analysis of the substrate determinants for the inosine recognition and cleavage

**Figure 3:**
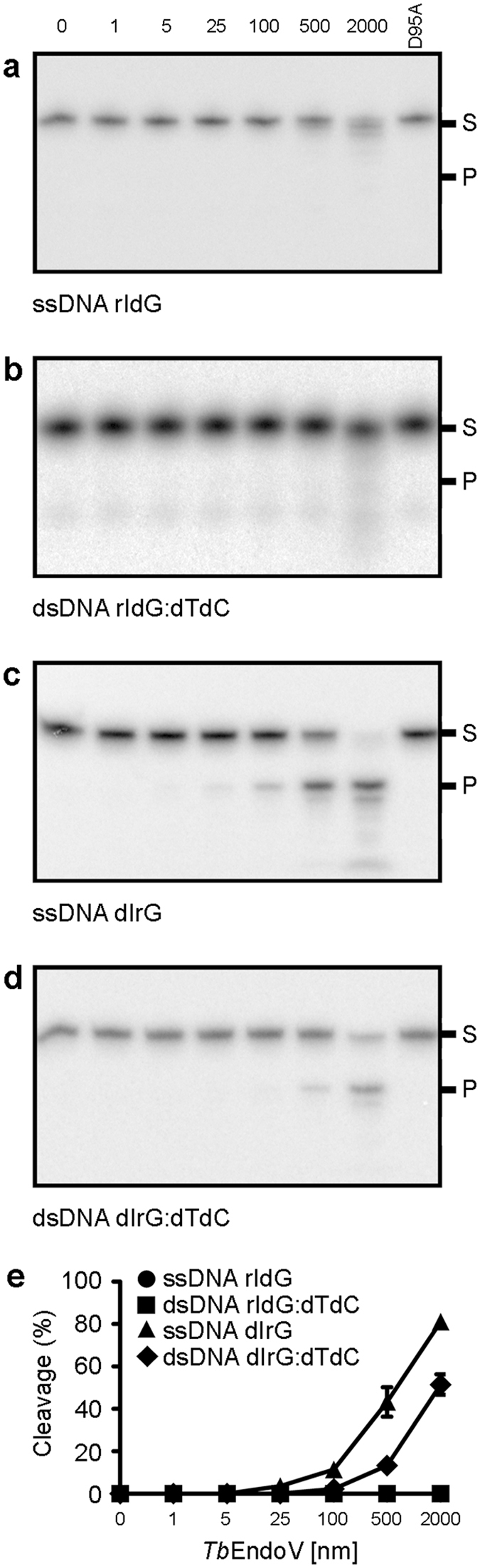
Analysis of substrate determinants for the incision activity of *Tb*EndoV. DNA substrates with (**a**,**b**) riboinosine or (**c**,**d**) a ribonucleotide located 3′ to the inosine lesion were incubated with increasing amounts of *Tb*EndoV (0–2000 nM, as indicated). (**e**) Plot of the percentage of substrate cleaved by endonuclease V activity versus total amount of protein used in the reaction. Each point represents the mean (±s.d.) from three independent experiments. The D95A catalytic mutant was also included as control. Reaction products were analyzed by 20% denaturing PAGE. Positions of the reaction substrate (S) and product (P) are indicated.

Both mammalian and prokaryotic EndoVs recognize inosine and cleave at the second phosphodiester bond 3′ to the lesion^14, 19, 20^. However, while human and mouse enzymes act only upon RNA, bacterial EndoV operates efficiently on DNA and RNA. The strong preference of trypanosomal EndoV for inosine in RNA suggests that the 2′ hydroxyl group of the ribose is required for an efficient catalysis. Here, we have investigated the effect of specific substrate determinants on *Tb*EndoV catalytic function. A set of DNA oligonucleotides was designed to examine how the presence of a ribose either at the lesion site or at the 3′-adjacent nucleotide affected the incision of *in situ* deamination products. As shown in Fig. 3, DNA substrates (ssDNA rIdG and dsDNA rIdG:dTdC) containing a riboinosine but followed by a deoxyribose are not efficiently cleaved by *Tb*EndoV (Fig. 3a,b,e). However, when a ribonucleotide is placed at the 3′ position of a deoxyinosine lesion, *Tb*EndoV incision activity is greatly enhanced, regardless the DNA substrate is single (ssDNA dIrG) or double-stranded (dsDNA dIrG:dTdC) (Fig. 3c,d,e).

### *Tb*EndoV acts upon tRNA substrates containing inosine

**Figure 4:**
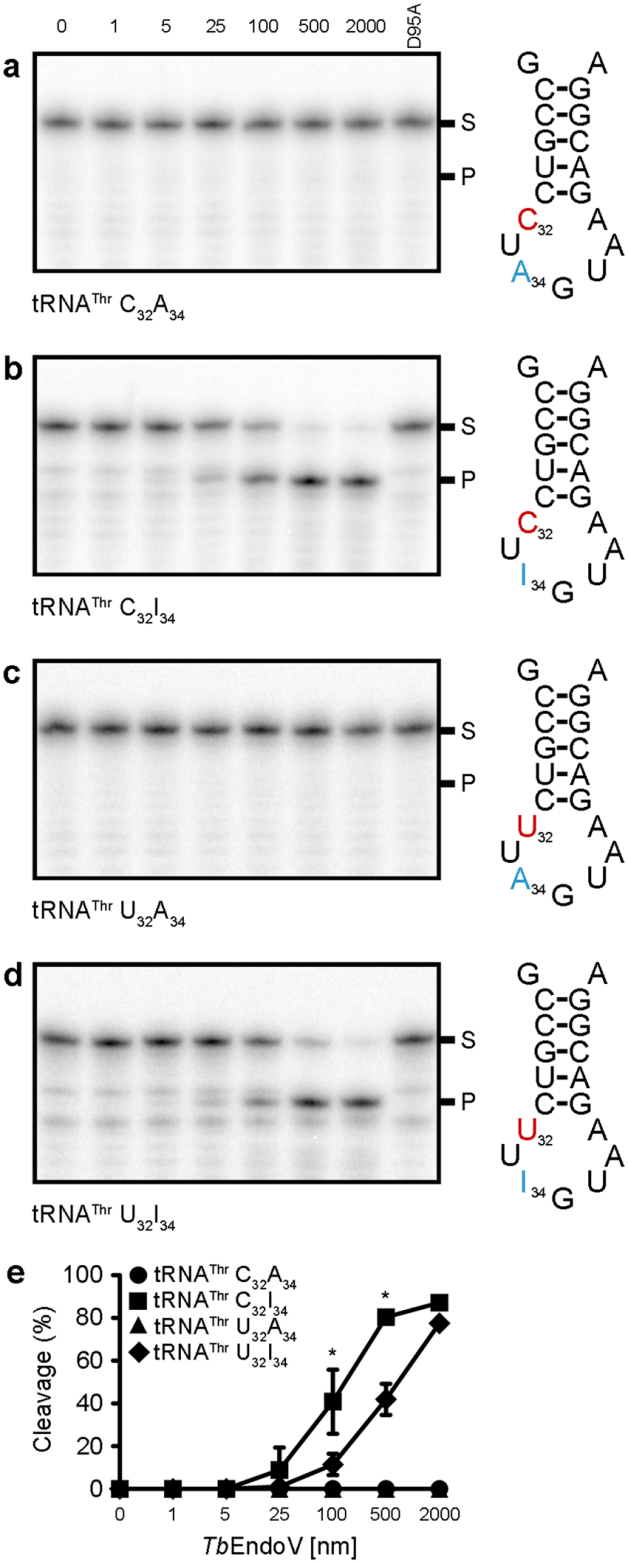
*Tb*EndoV can process tRNA-mimicking substrates containing inosine. Two positions (cytosine 32 and adenine 34) undergo editing in threonine-coding tRNAs in *T*. *brucei* ^4^. To replicate the edited substrates, bases corresponding to C32 and A34 were substituted by their corresponding deamination products, uracil and hypoxanthine respectively. Substrates were incubated with increasing amounts of *Tb*EndoV (0–2000 nM, as indicated). (**a**) Control non-edited substrate; (**b**) adenine-edited substrate; (**c**) cytosine-edited substrate and (**d**) double-edited substrate. (**e**) Quantification of the data from (**a**–**d**). Each point represents the mean (±s.d.) from three independent experiments. The asterisks show significant differences calculated by a two-tailed Student’s t-test: *p < 0.05. Reaction products were analyzed by 20% denaturing PAGE. Positions of the reaction substrate (S) and product (P) are indicated.

RNA-editing is a potential source of inosine in trypanosomes since ADAT proteins have been characterized in these organisms^4, 31^. In particular, threonine coding tRNA showed high editing levels at positions 32 (C-to-U editing) and 34 (A-to-I editing) in *T*. *brucei* ^4^. To determine whether this could be a biologically relevant RNA substrate, we assayed the *Tb*EndoV incision activity over non-edited, single- and double-edited tRNA-mimicking substrates (Fig. 4). As expected, *Tb*EndoV did not cleave the non-edited (Fig. 4a,e) or the C to U edited substrates with adenine at position 34 (Fig. 4c,e). In contrast, the tRNA containing Hx at position 34 (C_32_I_34_) was efficiently processed *in vitro* (Fig. 4b,e) and could be a potential cellular target for endonuclease V in *T*. *brucei*. Notably, the additional presence of U at position 32 had a negative impact on the endonuclease ability to cleave this tRNA substrate (U_32_I_34_), suggesting that cytosine deamination at position 32 may act as an inhibitory mechanism to prevent the processing of A to I-edited tRNAs by EndoV.

### Subcellular localization of *Tb*EndoV

**Figure 5:**
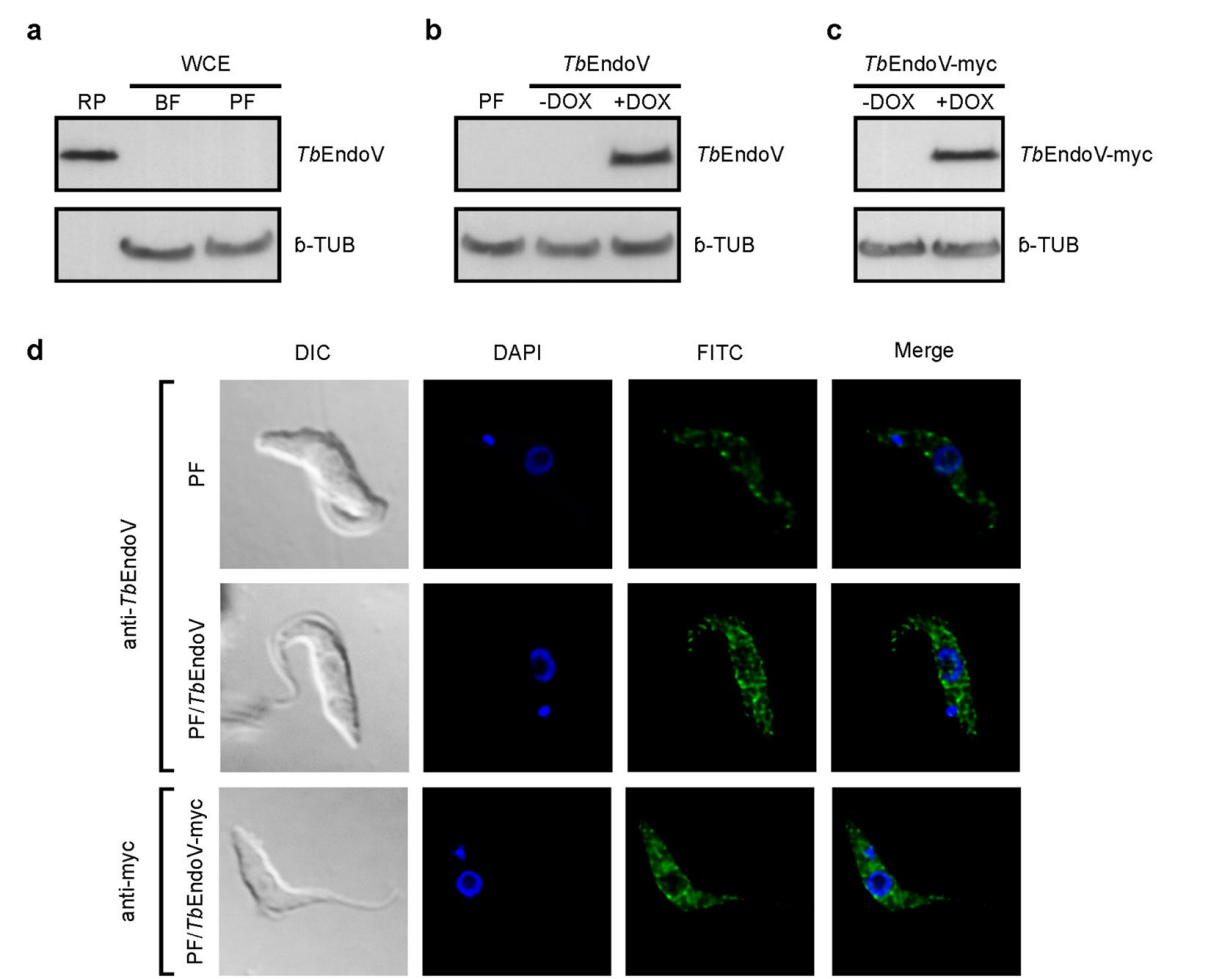
Subcellular localization of endonuclease V in *T*. *brucei* cells. (**a**) Analysis by western blot of the expression levels of EndoV in bloodstream (BF) and procyclic (PF) forms using an anti-*Tb*EndoV polyclonal antibody generated in our laboratory. Recombinant purified *Tb*EndoV (20 ng) (RP) was included as positive control. (**b**) Expression of native *Tb*EndoV and (**c**) *Tb*EndoV-myc was detected in doxycycline induced *Tb*EndoV- and *Tb*EndoV-myc-overexpressing PF cells using the anti-*Tb*EndoV polyclonal antibody or an anti-myc monoclonal antibody, respectively. Anti-β-tubulin was used as loading control in all western blotting analysis. (**d**) Immunofluorescence microscopy analysis showing the subcellular localization of *Tb*EndoV. Parental cell line (PF) (upper row), *Tb*EndoV- (middle row) and *Tb*EndoV-myc-overexpressing PF cells (lower row).

Endonuclease V has been localized to the cytosol of human cells^23^ but also associated to the nucleoli^32^. In order to determine the subcellular localization of the parasite enzyme, specific rabbit polyclonal antibodies against *Tb*EndoV were raised using soluble recombinant His-*Tb*EndoV as antigen. To ensure higher specificity, antibodies were purified from blood serum by affinity chromatography against the same pure recombinant protein. Immunoblotting analysis failed to detect EndoV protein in procyclic and bloodstream whole cell extracts, even though the antibody does react efficiently with twenty nanograms of purified recombinant protein (Fig. 5a), suggesting that EndoV expression levels are extremely low in trypanosomes. To overcome this limitation for subcellular localization studies, we generated procyclic *T*. *brucei* cell lines stably overexpressing *Tb*EndoV (PF/*Tb*EndoV) or a C-terminal Myc-tagged version of *Tb*EndoV (PF/*Tb*EndoV-myc). Protein levels were monitored using the purified anti-*Tb*EndoV and monoclonal anti-myc antibodies, respectively (Fig. 5b,c). Both, polyclonal and monoclonal antibodies, recognize a single and highly intense band in western blots. In spite of the high levels of protein synthesis, *Tb*EndoV overexpression did not appear to have any impact on proliferation and therefore these transgenic cells were considered suitable for cellular localization studies.

Immunofluorescence microscopy images obtained from the parental procyclic cell line (PF) (Fig. 5d, upper row) and the *Tb*EndoV-overexpressing cell line (middle row) revealed a granular staining pattern with predominant localization in the cytosol and at a much lesser extent in the nucleus. The same distribution pattern was verified in an independent experiment using a monoclonal anti-myc antibody on PF/*Tb*EndoV-myc cells (Fig. 5d, lower row). These results demonstrate that *Tb*EndoV performs an important function in the cytoplasm although a certain role in the nucleus cannot be excluded. Our analysis provides the first experimental evidence of the subcellular localization of a eukaryotic EndoV under endogenous expression conditions, avoiding this way potential overexpression-induced artifacts.

### *Tb*EndoV depletion impairs proliferation of *T*. *brucei* procyclic forms

**Figure 6:**
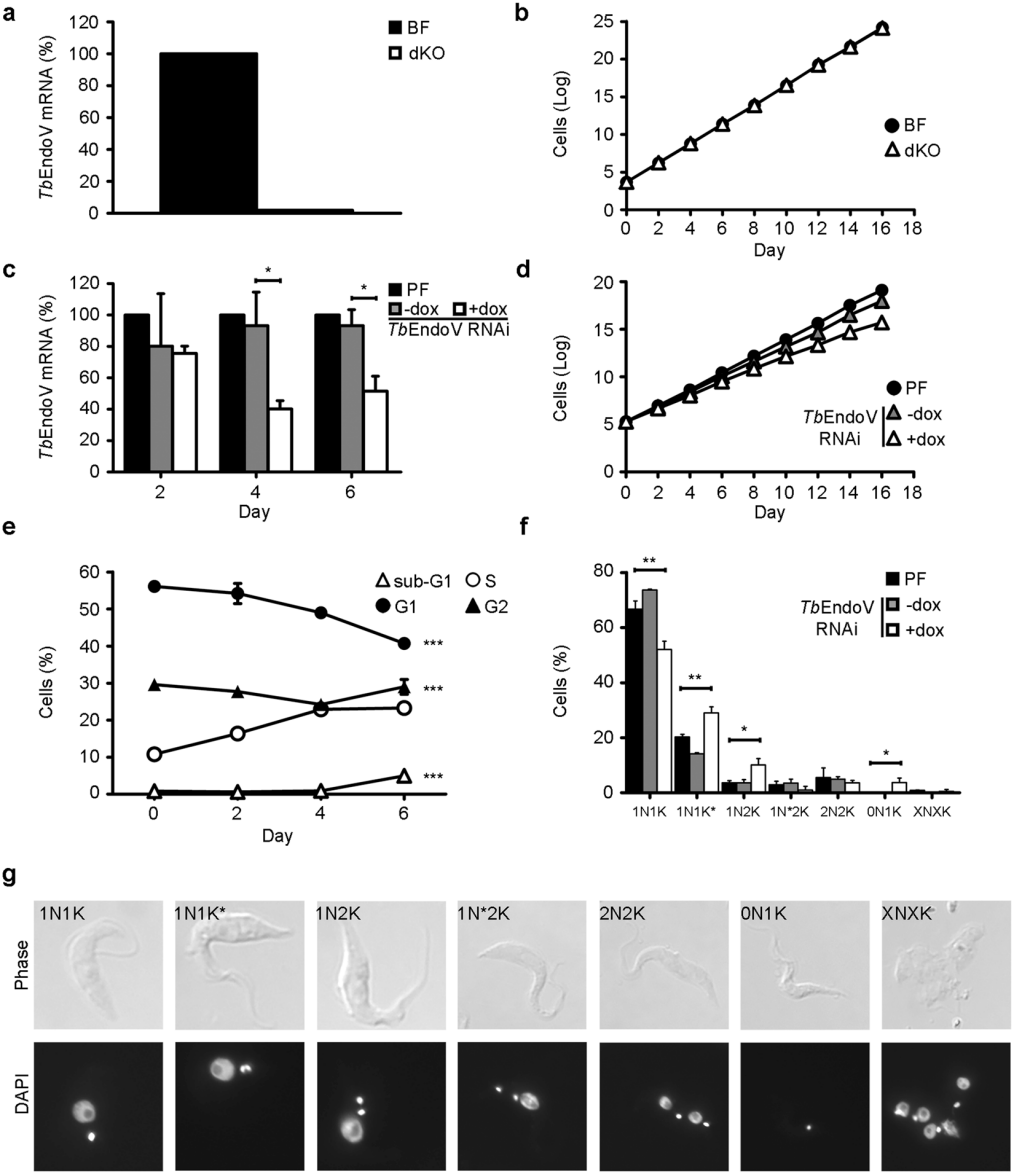
Characterization of *T*. *brucei* cell lines defective in endonuclease V. (**a**,**b**) *Tb*EndoV was knocked-out in bloodstream *T*. *brucei* cells. (**a**) *TbEndoV* mRNA levels and (**b**) proliferation curve are shown. (**c**,**d**) *Tb*EndoV depletion in procyclic *T*. *brucei* by RNA interference. (**c**) *TbEndoV* mRNA levels were monitored by quantitative-PCR at different time points after RNAi induction. qRT-PCR data correspond to the mean (±s.d.) of three samples from three independent experiments. The asterisks show significant differences calculated by a two-tailed Student’s t-test: *p < 0.05. (**d**) Plots showing the proliferation curves after RNAi induction. (**e**) Cell cycle progression was analyzed during a time course of 6 days using FACS in *Tb*EndoV-RNAi cells induced with 1 µg·mL^−1^ of doxycycline. Data correspond to the mean (±s.d.) of two samples coming from three independent experiments. Two-tailed Student’s t-test was calculated for differences between day 0 and 6 (***p < 0.001) (**f**) Quantification of nuclei (N) and kinetoplasts (K) in parental and *Tb*EndoV-RNAi cells after 6 days of induction with 1 µg·mL^−1^ of doxycycline. Data correspond to mean percentages (±s.d.) of total cells (>300) coming from three independent experiments. The asterisks show significant differences calculated by a two-tailed Student’s t-test: *p < 0.05, **p < 0.01, ***p < 0.001. (**g**) Representative fluorescence microscopy images of *Tb*EndoV-deficient cells at day 6 post-RNAi induction. Parasite morphology (Phase) and the number of nuclei (N) and kinetoplasts (K) are shown (DAPI staining). N* indicates an enlarged nucleus and K* an enlarged kinetoplast.

In order to assess whether *T*. *brucei* requires the EndoV activity for normal proliferation and survival, we proceeded to generate *EndoV*-null mutants in bloodstream and procyclic forms of the parasite. For bloodstream forms, replacement cassettes contained a puromycin-resistance gene (*PAC*) and a phleomycin-resistance gene (*BLE*), flanked by 550 bp of the *EndoV* 5′-UTR and 3′-UTR sequences (Fig. S3). All DNA fragments were obtained by PCR amplification of *T*. *brucei* 427 genomic DNA and primers were designed from the sequence obtained from the GeneDB database. Linear targeting fragments containing each of the inactivation cassettes were initially transfected into *T*. *brucei* bloodstream strain yielding recombinant clones, which were efficiently isolated in selective media with puromycin or phleomycin. One of the puromycin-resistant clones was subjected to a second round of inactivation with the *BLE* cassette obtaining this way transfectants resistant to puromycin and phleomycin. Integration of the *PAC* or *BLE* markers was monitored by PCR using primers that hybridize with the flanking genes upstream and downstream from the *TbEndoV* gene in combination with marker-specific primers (Fig. S3). As expected, no expression of *TbEndoV* mRNA was observed by RT-PCR in the selected clone (Fig. 6a). Despite lacking *Tb*EndoV, double knockout cells exhibited similar proliferation rate as parental cells (Fig. 6b), providing direct evidence that the enzymatic activity of *Tb*EndoV is not essential for viability or proliferation of bloodstream trypanosomes *in vitro*.

Inactivation of *Tb*EndoV by homologous recombination was also attempted in procyclic *T*. *brucei* cells; however, two-allele replacement could not be achieved after several attempts. We therefore used an alternative knock-down approach that allows monitoring the cellular events that follow the reduction of EndoV activity. We therefore proceeded to silence *Tb*EndoV expression using the RNA interference (RNAi) methodology. Due to the low constitutive expression of *Tb*EndoV, we monitored the inhibition of expression using qRT-PCR. As shown in Fig. 6c, *Tb*EndoV could be successfully depleted in doxycycline-induced RNAi-transfected cells. Determination of the *TbEndoV* mRNA levels at two, four and six days after doxycycline addition revealed a decrease of 25%, 60% and 49% respectively, in comparison to the parental strain. A time course analysis of parasite growth was carried out during 16 days to determine the effect of *Tb*EndoV depletion. Cells where *Tb*EndoV was down-regulated exhibited a moderate but consistent defect in proliferation with respect to non-induced or parental control cells (Fig. 6d).

EndoV-deficient cells were further analyzed by quantifying the DNA content by flow cytometry together with DAPI staining of nuclei and kinetoplasts. FACS analysis revealed a significant increase of EndoV-deficient trypanosomes in the S-phase (Fig. 6e), which is consistent with the accumulation of cells with an enlarged kinetoplast (1N1K*) observed in cells that have already initiated nuclear DNA duplication (Fig. 6f,g). At the same time, cells in which EndoV was knocked-down also displayed a decrease in 1N1K (corresponding to cells in G1) and a concomitant increase in the population of 1N2K cells (G2 phase) (Fig. 6e,f). The major anomaly was the presence of cells with one kinetoplast but without nucleus (0N1K), also known as zoids (Fig. 6f,g), that may account for the sub-G1 population detected by flow cytometry. Similar cell phenotypes have been described in procyclic *T*. *brucei* cells after treatment with aphidicolin or bleomycin where the inhibition of nuclear S phase was followed by defective mitosis^33, 34^.

### *Tb*EndoV localizes to stress granules after starvation

**Figure 7:**
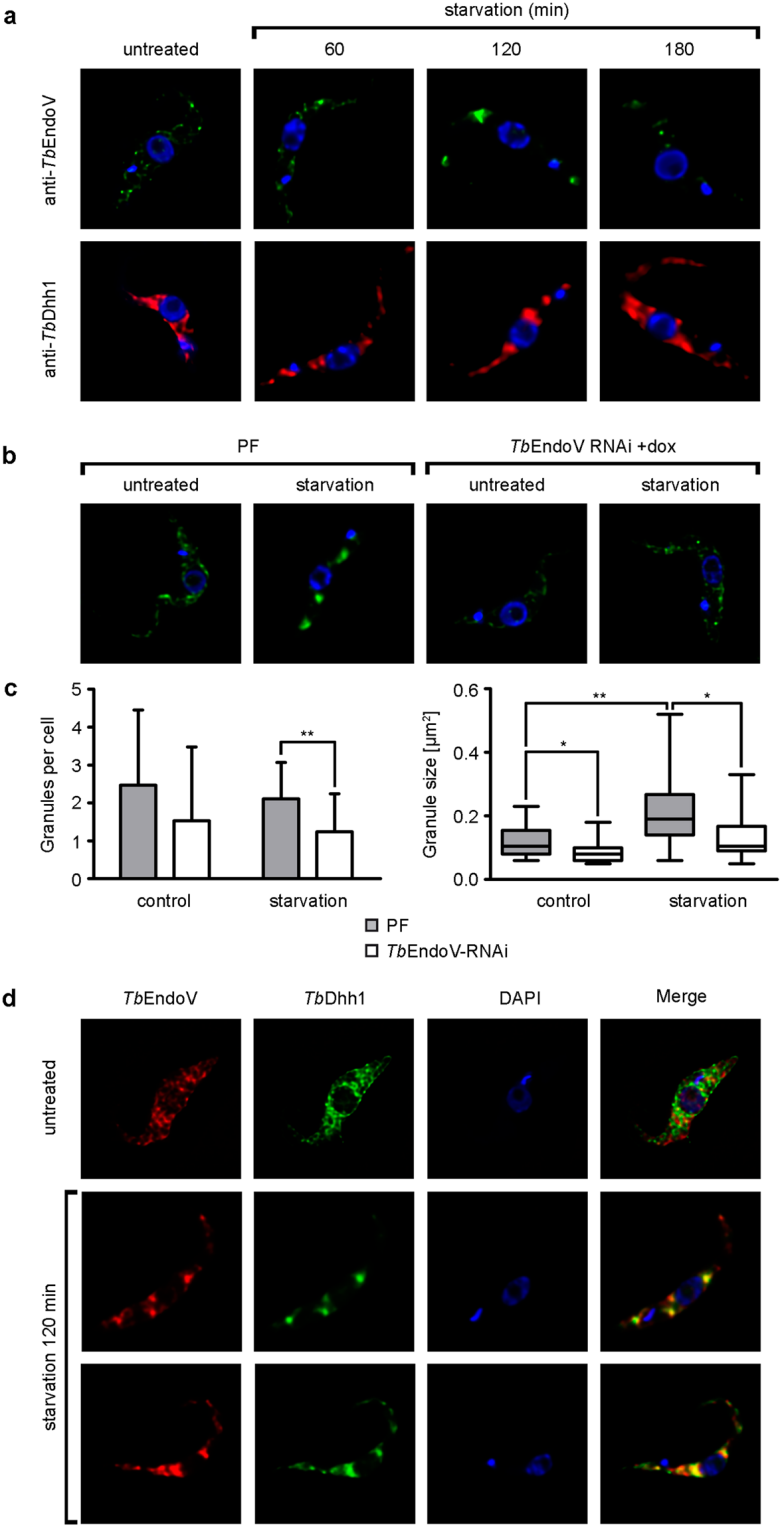
*Tb*EndoV relocalizes into cytoplasmic granules upon nutritional stress. (**a**) To induce nutritional stress, trypanosomes were washed and maintained in PBS for different time periods as indicated prior to fixation, and then processed for single immunofluorescence staining using the following pairs of primary and secondary antibodies: rabbit anti-*Tb*EndoV/Alexa Fluor® 488-conjugated goat anti-rabbit IgG or rabbit anti-*Tb*Dhh1/Alexa Fluor® 488-conjugated goat anti-rabbit IgG. Untreated (no exposure to PBS) cells were used as control. Nuclear DNA and the kinetoplast were visualized by DAPI staining. The images were representative of each staining from at least two independent experiments. (**b**) Parental PF and *Tb*EndoV-RNAi cells under normal (untreated) or starvation-induced (120 min PBS) conditions were immunostained with rabbit anti-*Tb*EndoV and Alexa Fluor® 488-conjugated goat anti-rabbit IgG. (**c**) Comparative analysis of *Tb*EndoV relocalization in control and RNAi-silenced trypanosomes. *Left*, number of granules per cell in normal or starvation conditions in the wild type or *Tb*EndoV-RNAi procyclic cells. Data are shown as mean (±s.d). At least 16 cells were analyzed for each group. *Right*, median representation of the mean granule area per cell: box represents the interquartile range (IQR) and whiskers are maximum and minimum values. Differences in granule areas are statistically significant between untreated and starvation-induced PF cells (p = 0.002) and between PF and *Tb*EndoV-RNAi cells in normal growth conditions or under starvation (p = 0.03). Unpaired two-tailed Student’s *t*-test with Welch’s correction; *p < 0.05 **p < 0.01. (**d**) Colocalization of *Tb*EndoV and *Tb*Dhh1 in starvation stress granules. Starvation stress was induced by maintaining the trypanosomes in PBS for 120 min prior to fixation. Cells were then processed for double immunofluorescence staining using the following pairs of primary and secondary antibodies: Alexa Fluor® 594-conjugated rabbit anti-*Tb*EndoV and rabbit anti-*Tb*Dhh1/Alexa Fluor® 488-conjugated goat anti-rabbit IgG. Untreated (no exposed to PBS) cells were used as control. Nuclear DNA and the kinetoplast were visualized by DAPI staining. The images were representative of each staining from at least two independent experiments.

Human EndoV has been shown to relocate to cytoplasmic RNA-containing granules in response to cellular stress caused by sodium arsenite where it could be contributing to the elimination of damaged inosine-containing transcripts^24^. Stress granules (SGs) are transient aggregates of stalled translation initiation complexes whose function is to preserve the mRNAs and prevent their translation until the normal conditions are restored. Oxidative stress, glucose deprivation, heat-shock, viral infections etc. are demonstrated inducers of stress granule formation^35^. In trypanosomes, SGs have been observed in the procyclic forms that have to cope with periods of starvation and temperature changes inside the insect^36^. To address whether *Tb*EndoV might be relocalizing in SGs after starvation-induced stress, cells were cultured in carbon-free buffer (PBS) for different time periods and subsequently monitored by fluorescence microscopy (Fig. 7a). After 120 min in PBS, endogenous *Tb*EndoV protein accumulated into discrete *foci* which were bigger in size than the granule particles observed in cells grown in normal rich medium (untreated) (Fig. 7c). Under the same conditions, the DEAD-box RNA helicase *Tb*Dhh1, a well-characterized component of starvation SGs^37^, also relocates into *foci* although with different kinetics, thus validating the protocol used to induce starvation (Fig. 7a). To validate the specificity of the signal detected, cells where *Tb*EndoV expression was silenced by RNAi were analyzed by immunofluorescence microscopy (Fig. 7b). Quantitative analysis revealed that trypanosomes expressing low levels of *Tb*EndoV exhibited a significant reduction in the number and size of *Tb*EndoV-positive granules after 120 min in the absence of a carbon source thus confirming the specific accumulation of this endonuclease in starvation-induced granules (Fig. 7c).

We next proceeded to establish whether *Tb*EndoV does actually colocalize with the RNA-binding protein *Tb*Dhh1 in SGs upon starvation. To perform the colocalization experiments, the anti-*Tb*EndoV antibody was non-covalently labeled with Alexa Fluor® 594 while the polyclonal anti-*Tb*Dhh1 was detected with an Alexa Fluor® 488-conjugated secondary anti-rabbit antibody. Immunostaining showed extensive colocalization of *Tb*EndoV and *Tb*Dhh1granules at 120 minutes after induction of starvation (Fig. 7d). Pearson’s correlation and Manders’ overlap coefficients were determined to establish the degree of colocalization. Manders’ coefficient indicate that 74% of the signal corresponding to *Tb*Dhh1 overlaps with *Tb*EndoV (M1 = 0.74 ± 0.13) and *vice versa*, 70% of *Tb*EndoV fluorescence ovelaps with *Tb*Dhh1 (M2 = 0.70 ± 0.11). The colocalization is also supported by the Pearson’s correlation coefficient (0.87 ± 0.04). These data demonstrate the presence of *Tb*EndoV in SGs and a role for this protein in the stress response to starvation.

## Discussion

*Trypanosoma brucei* is an extracellular parasite under constant exposure to oxidative and nitrosative stress either generated as a byproduct of its own metabolism or produced by immune effector cells in response to parasite infection^38, 39^. It is well established that ROS and RNS can damage vital molecules, such as proteins, lipids, or nucleic acids^40^. Therefore, protozoan survival and genomic stability greatly depends on DNA repair systems, which for that reason could be excellent targets for chemotherapeutic interventions^41^.

Deaminated DNA bases such as uracil and hypoxanthine arise as result of spontaneous hydrolysis or induced by oxidative or nitrosative agents. These lesions are the substrate of specialized DNA glycosylases as part of the BER system, which carries out the correction of DNA alterations that do not distort the overall structure of DNA helix. *T*. *brucei* expresses a single uracil-DNA glycosylase activity (UNG)^42^ responsible for excision of U but no glycosylases with capacity to excise Hx, generally an alkylpurine-DNA glycosylase^43^, have been identified in kinetoplastid parasites^44^. While in bacteria, EndoV provides a back-up mechanism for the repair of Hx and U^45^, trypanosomal EndoV is not endowed with significant activity over DNA substrates and therefore it is unlikely that this enzyme performs an important role in DNA repair. Nevertheless, we cannot exclude the possibility that neglected factors in our *in vitro* cleavage assay may enhance the enzyme activity over DNA *in vivo*. Similarly to mammalian EndoVs which have been associated to a possible role in RNA processing^19, 23^, *Tb*EndoV exhibits strict specificity for inosine in RNA suggesting that this might be the potential *bona fide* substrate in physiological conditions.

Several potential biological targets can be anticipated for *Tb*EndoV. Deamination of adenine can occur in RNA and their precursors via hydrolytic and nitrosative reactions upon exposure to endogenous or exogenous agents^46^. *T*. *brucei* is exposed to RNS in the early steps of an infection, after nitric oxide (NO) and ROS are released by phagocytes as part of the primary immune response^47^. RNS can react with nucleic acids generating strand breaks and base modifications including deaminations^48^, which constitute the primary cause of NO-induced mutagenicity in a variety of organisms^49, 50^. The nucleotide pool may also undergo base deaminations under the same circumstances leading to the generation of non-canonical ITP that could be erroneously incorporated by the RNA polymerase during transcription. Supporting this notion, *E*. *coli* and yeast cells defective in inosine triphosphate pyrophosphatase (ITPase) exhibit increased levels of inosine in their RNA^51^. The presence of Hx in mRNAs could have mutagenic consequences during gene expression since it is recognized as guanine during protein translation^5^. Likewise, the presence of inosine in tRNAs and rRNAs might modulate or interfere with protein translation as described for other modifications^52^. Recognition and cleavage by EndoV endonuclease activity of adenine-deaminated transcripts would prevent the formation of mutant proteins thus acting as a mechanism to eliminate potentially harmful mRNAs.

Another source of inosine in RNA relies on the enzymatic activity of specific adenosine deaminases acting on mRNA (ADAR) and tRNA (ADAT)^1^. These enzymes play an important role introducing inosine in specific positions of RNA, a key process for correct splicing and codon diversity^53^. While no ADAR protein has been identified in *T*. *brucei*, adenine deamination in tRNAs by ADAT enzymes has been extensively described^4, 31^. In particular, a heterodimer made up of two subunits (ADAT2 and ADAT3) is responsible for specific A to Hx deamination^3^ in *T*. *brucei*. Eight different tRNA species (for Ala, Arg, Ile, Leu, Pro, Ser, Thr and Val amino acids) have adenine at the wobble position of the anticodon and therefore, potential A-to-I editing sites. Indeed, threonine coding tRNA is highly edited at position 34 (A-to-I) but also at position 32 (C-to-U) in *T*. *brucei* ^4^. *In vitro*, *Tb*EndoV cleaves the trypanosomal A-to-I edited tRNA substrates with high efficiency, suggesting that the turnover of edited tRNAs may be initiated by this inosine-specific endonuclease activity. The presence of U at position 32 was somewhat detrimental for activity, which suggests that EndoV-mediated cleavage may be modulated by editing events.

Beyond a potential role in tRNA degradation, *Tb*EndoV ability to cleave tRNAs is reminiscent of the endonucleolytic activity of yeast Rny1 and mammalian ribonuclease angiogenin. These nucleases carry out the incision of tRNAs within the anticodon loop generally as part of a stress response but in this case, tRNA cleavage is not intended to promote tRNA degradation since full-length tRNA levels are not substantially affected. Instead, tRNA-derived small RNAs themselves may function to inhibit translation or promote the degradation or repression of specific mRNAs under particular physiological conditions^54, 55^. The presence of half-tRNA molecules have been detected in *Tetrahymena*, *Aspergillus* and *Trypanosoma cruzi* following nutritional stress^56–58^ while *Saccharomyces*, *Arabidopsis* and human cells accumulate cleaved tRNAs in response to oxidative stress^59^. Interestingly, *T*. *cruzi* secretes extracellular vesicles containing tRNA halves that may reach host mammalian cells and alter the expression of genes involved in the immune response in order to facilitate the infection^60^. Although the form of tRNA processing is essentially different from the more specific endoV-mediated cleavage of A-to-I edited tRNAs, it raises the question as to whether cleaved tRNAs generated by the action of *Tb*EndoV might perform analogous functions and participate in non-canonical regulatory pathways.

Subcellular localization analysis revealed that *Tb*EndoV is primarily a cytosolic enzyme. Likewise, human EndoV has been localized to the cytosol but also associated to the nucleoli, suggesting an involvement in ribosomal RNA metabolism^23, 32^. Furthermore, arsenite-induced oxidative stress induces the accumulation of hEndoV in cytoplasmic granules where it colocalizes with polyadenylate-binding protein C1 (PABPC1)^24^. In procyclic trypanosomes, *Tb*EndoV colocalizes with *Tb*Dhh1 in cytoplasmic stress granules upon starvation. Similarly to sodium arsenite, glucose deprivation also induces ROS production which in turn may cause harmful base deaminations in mRNAs^61^. While SGs are mainly involved in storage and regulation of translation of mRNAs, Pbodies contain mRNAs targeted for degradation as well as factors involved in mRNA turnover. These granules are not hermetic entities neither in mammalian cells, where SGs may transiently establish physical and functional interactions with P bodies^35^, nor in *T*. *brucei* where SGs and P bodies share several of their components including *Tb*Dhh1^62^. Likewise, it has been suggested that in *T*. *brucei*, some of the stress granules might arise from preexisting P bodies as described for yeast^63^. Although whether *Tb*EndoV is a constitutive component of P bodies remains unclear, its presence in starvation-induced SGs suggest that it could be performing quality control of deaminated mRNAs and regulating the transfer of damaged transcripts to adjacent P bodies for their elimination.

We have shown that suppression of *Tb*EndoV activity impairs the proliferation *in vitro* of procyclic parasites whereas bloodstream trypanosomes can still grow normally. While it has been reported that around 6% of the genes are differentially expressed between procyclic and bloodstream forms of *T*. *brucei* ^64^, *Tb*EndoV appears to be equally expressed in both stages of the parasite^64, 65^ and thus different enzyme requirements would not explain the obtained phenotype. It is possible that *Tb*EndoV is involved in the homeostasis of specifically edited RNAs or establishes interactions with procyclic-specific proteins. Another potential factor accounting for a distinct role of *Tb*EndoV in procyclics may relate to the major differences in mitochondrial metabolism between mammalian and insect-stage parasites. The identification of specific cellular substrates of the enzyme and its role in the occurrence of inosine-containing RNA will contribute to the understanding of the relevance of *Tb*EndoV in parasite survival.

In summary, the data presented in this work show that *Tb*EndoV exhibits incision activity preferentially upon inosine-containing RNA and appears to be needed for normal proliferation of procyclic forms where it could play a critical role in the metabolism of RNAs that are specifically required or expressed in this life stage of the parasite.

## Methods

### Expression and purification of wild-type and mutant endonuclease V

The endonuclease V coding sequence (Tb927.10.6860) was amplified from *T*. *brucei brucei* (Lister 427) genomic DNA using specific primers 5′-GCG AAT TCA TGG GTG TGG GAG AAA TGA GT-3′ and 5′-GCG GAT CCT CAG CAT TCA TTT AGT TGA ATC-3′. The PCR product was cloned into pMAL-c2X vector that allows protein expression in fusion to Maltose-Binding Protein (MBP) at the N-terminus (New England Biolabs). A *Tb*EndoV-D95A mutant sequence was generated using QuikChange Lightning Multi Site-Directed Mutagenesis Kit (Agilent Technologies) with the following primers: 5′-C CAC GTC GGC GGC GTT GCC ATA TCC TTT ATT CAG G-3′ and 5′-C CTG AAT AAA GGA TAT GGC AAC GCC GCC GAC GTG G-3′ (underlined characters indicate the codon change). MBP-EndoV proteins were purified by amylose affinity (New England BioLabs) followed by gel filtration chromatography. Fractions containing MBP-EndoV were pooled, dialyzed against storage buffer [10 mM Tris-HCl, pH 7.5, 200 mM NaCl, 1 mM DTT and 20% glycerol] and stored at −80 °C.

### Enzymatic assays

The 21-mer oligonucleotide substrates containing modified bases were synthesized by TriLink Biotechnologies. Oligonucleotide sequences used in all assays can be found in supplementary material (Fig. S1). All substrates were labeled at the 5′-end with [γ-^32^P]ATP (3000 Ci/mmol; Perkin Elmer) and T4 polynucleotide kinase (New England Biolabs) and purified using MicroSpin G25 columns (GE Healthcare).

In a standard reaction (10 µL final volume), 5 fmol of [γ-^32^P]-labeled substrate were incubated in reaction buffer [20 mM Tris-HCl pH 7.5, 1 mM MnCl_2_, 1% BSA, and 10 units of RNasin^®^ Plus Ribonuclease Inhibitor (Promega) for RNA substrates] with increasing enzyme amounts for 1 hour at 37 °C. Reactions were terminated by adding 10 µL of loading buffer (90% formamide, 0.1% bromophenol blue, 0.1% xylene cyanol and 50 mM EDTA) and heating at 95 °C for 5 min. Reaction products were resolved on denaturing 20% (w/v) polyacrylamide gel electrophoresis (PAGE) (19:1 acrylamide/bisacrylamide). Gels were scanned in a Typhoon 9400 scanner (Amersham Biosciences) using a Storage Phosphor Screen (Amersham Biosciences). Gel analysis to determine the percentage of cleavage was performed with Fiji software (ImageJ 1.51d).

### Antibody generation

To obtain a polyclonal anti-*Tb*EndoV antibody, a rabbit was immunized using soluble purified recombinant *Tb*EndoV. Before injecting the antigen into the rabbit, 450 µg of pure protein diluted in PBS was mixed with Freund’s adjuvant (1:1 ratio). Antibody-containing serum was collected and affinity-purified using pure recombinant protein coupled to Affi-Gel^®^15 resin (BioRad) following the manufacturer’s instructions.

### Trypanosome growth and transfection

All cell lines used in this work derive from the *Trypanosoma brucei brucei* single marker bloodstream form (BF)^66^ and the *Trypanosoma brucei brucei* procyclic cell line 449 (PF)^67^. Bloodstream cells were cultured at 37 °C and 5% CO_2_ in HMI-9 medium supplemented with 10% (v/v) of fetal bovine serum (FBS). Procyclic parasites were cultured at 28 °C in SDM-79 medium with 10% FBS and 7.5 µg·ml^−1^ Haemin.

Trypanosomes were transfected by electroporation in Cytomix medium for bloodstream cells and Zimmerman medium for procyclic cells, as previously described^66, 68^. Clones were selected with appropriate selection drugs at the following concentrations: puromycin (Sigma), 0.1 µg·mL^−1^ (BF) and 1 µg·mL^−1^ (PF); hygromycin (Sigma), 5 µg·mL^−1^ (BF) and 50 µg·mL^−1^ (PF); phleomycin (Sigma), 2.5 µg·mL^−1^ (BF) and blasticidin (Invitrogen), 10 µg·mL^−1^ (PF). RNAi and *Tb*EndoV expression were induced using 1 µg·mL^−1^ of doxycycline (Sigma).

### Knock-out, RNAi and overexpression constructs

Primers sequences were obtained from GeneDB (Tb927.10.6860) and PCR amplifications were made using *T*. *brucei* 449 genomic DNA as template. In order to generate *Tb*EndoV knockout cells, both alleles were replaced by resistance markers. Replacement cassettes were designed with resistance genes for phleomycin, blasticidin or puromycin flanked by 500 base pairs of the 3′ and 5′ *Tb*EndoV UTRs. The 5′-UTR was amplified with primers 5′-GCG GAT CCC AAT CGT ATT TGC CCA TTT TAT TCT GAC ACA C-3′ (forward) and 5′-ATG AAT TTC AGA AGA CCT TGC TGT GAT GTT CAG TTC CTG TGT TTA-3′ (reverse), and the 3′-UTR with primers 5′-GCG GCC ACG GGA CGT TGA ACG CTT GTT TC-3′ (forward) and 5′-GCG GAT CCG GGA GTA CAG GGA CCA GAG TAC CC-3′ (reverse). BamHI restriction sites were included (underlined) to allow the linearization of the plasmid.

For the RNA interference construct, a region of *Tb*EndoV of 400 bp (position 29–428 of the coding region) was amplified using the following set of primers: (forward) 5′-GCG GAT CCA AGC TTC CGC GAT AAA GTT GGA CAT G-3′ and (reverse) 5′-AAC GGG CCC GCC TCC CGG AAC GCG AGG-3′. The amplification product was cloned in pGR19^69^ as previously described^70^.

To overexpress native *Tb*EndoV, the coding sequence was amplified with primers 5′-GCC ATA TGG GTG TGG GAG AAA TGA GTG-3′ and 5′-GCG GAT CCT CAG CAT TCA TTT AGT TGA ATC-3′ including NdeI and BamHI restriction sites (underlined). The PCR product was cloned into pGRV23b expression vector^42^. To generate a construct for the expression of *Tb*EndoV fused to a C-terminal myc tag, the coding sequence lacking the stop codon was amplified using primers 5′-GCC ATA TGG GTG TGG GAG AAA TGA GTG-3′ and 5′-GCG TTA ACG CAT TCA TTT AGT TGA ATC CGT CGG A-3′ including NdeI and HpaI restriction sites (underlined). The PCR product was cloned into pGRV33 expression vector^42^.

Knockout and RNAi transfected cells were analyzed by monitoring *Tb*EndoV mRNA levels via quantitative PCR using the primers 5′-GAT GGT TGT GGC GTG CAC CAT CCA CTT C-3′ and 5′-GAC GGT TGG GCG TAG CCG CAT AAC C-3′. Myosin levels were measured using the primers 5′-CTG CAG AAC AAG CAC GGC ATT T-3′ and 5′-ACG CTC AAC AGT GGC AGT GGA A-3′.

### Immunofluorescence studies

Immunofluorescence (IF) studies were performed as previously described^70^. Briefly, parasites were harvested, washed twice in PBS (137 mM NaCl, 4 mM Na_2_HPO_4_, 1.7 mM KH_2_PO_4_ and 2.7 mM KCl), and fixed in 4% PFA in wash solution (PBS 1x, 0.2% Tween^®^ 20) on a poly-l-lysine coated slide for 30 min. Fixed parasites were washed once in wash solution and permeabilized and blocked with 1% of NP40 in blocking solution (wash solution containing 1% blocking reagent (Roche)) during 75 min. The IF was performed by incubation with either rabbit polyclonal anti-*Tb*EndoV (1:100) or mouse monoclonal anti-c-myc (Sigma, 1:100) primary antibodies in blocking solution. Alexa Fluor® 488 goat anti-rabbit IgG or anti-mouse IgG were used as secondary antibody (Sigma, 1:500), respectively. Preparations were finally mounted with ProLong^®^ Gold Antifade Reagent with DAPI (Life Technologies). Vertical stacks of up to 40 slices (0.2 µm steps) were captured using an Olympus wide-field microscope and Cell-R IX81 software. Images were deconvolved and pseudo-colored with Huygens Essential software (version 3.3; Scientific Volume Imaging).

For nutritional stress studies, 25 million log-phase parasites were washed twice in PBS, and starved by incubation in PBS during the indicated time. After starvation, IF was performed by incubation with rabbit polyclonal anti-*Tb*EndoV (1:100) or anti-*Tb*Dhh1 (1:100) primary antibodies in blocking solution. Alexa Fluor® 488 goat anti-rabbit IgG and Alexa Fluor® 594 goat anti-rabbit IgG were used as secondary antibody (Sigma, 1:500), respectively. To study *Tb*EndoV and *Tb*Dhh1 colocalization, anti-*Tb*EndoV antibody was labeled with Alexa Fluor® 594 using Zenon™ Rabbit IgG Labeling Kit (ThermoFisher) following manufacturer’s instructions. After incubation with both the anti-*Tb*Dhh1 primary antibody and Alexa Fluor® 488 goat anti-rabbit IgG, samples were incubated with an excess of a nonspecific primary antibody to block the secondary antibody. Preparations were then incubated with the Alexa Fluor® 594-conjugated anti-*Tb*EndoV (1:10) during 1 hour and fixed in 4% PFA in PBS for 15 minutes.

All images were analyzed using Fiji/ImageJ software. Degree of colocalization was determined using Pearson’s correlation coefficient and Manders’ overlap coefficient. Both coefficients were calculated with JACoP plugin, using a manual threshold for Manders’ coefficient, and given values are the mean of at least 18 cells. Overlapping of *Tb*Dhh1 over *Tb*EndoV is represented by M1, while overlapping of *Tb*EndoV over *Tb*Dhh1 is represented by M2. For the determination of the number and size of granule particles, a threshold of 0.05 microns^2^ and 35.000 of signal intensity was manually set over Z-projection cell images (using the sum slice method).

### Fluorescence-activated cell sorting (FACS) analysis and quantification of nuclei and kinetoplasts

PF cells (10^7^ cells per sample) were harvested by centrifugation and fixed with ethanol. FACS analysis with propidium iodide staining was carried out as previously described^70^. To determine the number of nuclei and kinetoplasts per cell, trypanosomes (10^6^ cells per sample) were harvested by centrifugation, fixed with paraformaldehyde and mounted with ProLong^®^ Gold Antifade Reagent with DAPI (Life Technologies). A Zeiss Axiophot microscope (Carl Zeiss, Inc.) was used to carry out microscopy and digital image acquisition. The quantification analysis was performed in individual cells from three independent experiments (n > 300).

### Data availability

No datasets were generated or analyzed during the current study.

## Electronic supplementary material

Supplementary Information
