# Supplementary material for: Insights into the role of endonuclease V in RNA metabolism in Trypanosoma brucei
Source: Sci Rep. 2017 Aug 17;7:8505. doi: 10.1038/s41598-017-08910-1 (PMC5561087; doi:10.1038/s41598-017-08910-1)
Supplement: Supplementary file 1 — Supplementary Information [file 41598_2017_8910_MOESM1_ESM.pdf]

Insights into the role of endonuclease V in RNA metabolism in *Trypanosoma brucei*

Daniel García-Caballero, Guiomar Pérez-Moreno, Antonio M. Estévez, Luis Miguel Ruíz-Pérez, Antonio E. Vidal* and Dolores González-Pacanowska*.

Description:	5' to 3' sequence:
DNA dI	CCT GCC CTG IGC AGC TGT GGG
DNA dU	CCT GCC CTG UGC AGC TGT GGG
RNA rI	CCU GCC CUG IGC AGC UGU GGG
DNA rIdG	CCT GCC CTG [rI]GC AGC TGT GGG
DNA dIrG	CCT GCC CTG I[rG]C AGC TGT GGG
Complementary DNA dT	CCC ACA GCT GCT CAG GGC AGG
Complementary DNA dC	CCC ACA GCT GCC CAG GGC AGG
Complementary DNA dG	CCC ACA GCT GCG CAG GGC AGG
Complemnetary DNA dA	CCC ACA GCT GCA CAG GGC AGG
Complementary RNA rU	CCC ACA GCU GCU CAG GGC AGG
Complementary RNA rC	CCC ACA GCU GCC CAG GGC AGG
$tRNA^{Thr}$ $C_{32}A_{34}$	GCC GUC CUA GUA AGA CGG A
$tRNA^{Thr}$ $C_{32}I_{34}$	GCC GUC CUI GUA AGA CGG A
$tRNA^{Thr}$ $U_{32}A_{34}$	GCC GUC UUA GUA AGA CGG A
$tRNA^{Thr}$ $U_{32}I_{34}$	GCC GUC UUI GUA AGA CGG A
ssRNA rI (2)	CCG UAG AGC UAC IGA UCG GUC ACC G

Supplementary Figure S1. List of the oligonucleotides used to characterize the endonuclease activity of *Tb*EndoV.

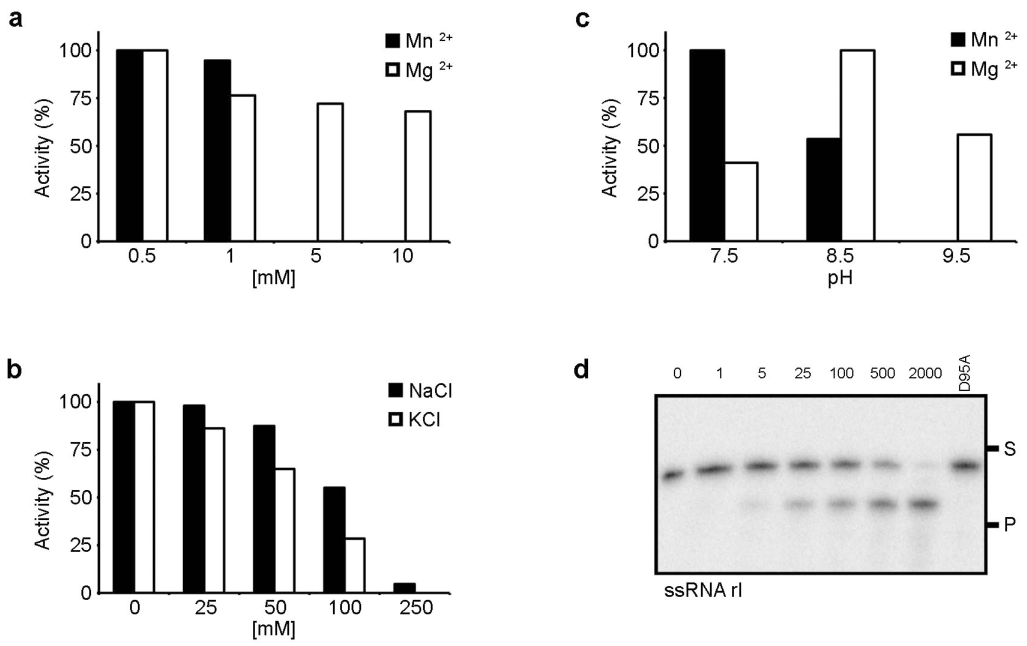

Supplementary Figure S2. Factors influencing *Tb*EndoV incision activity. Inosine-containing RNA substrates were incubated with 10 nM of *Tb*EndoV under different reaction conditions. (a) Increasing amounts of metal cofactors Mg^{2+} or Mn^{2+} (0.5 – 10 mM, as indicated). (b) Increasing amounts of NaCl or KCl (0 – 250 mM, as indicated). (c) Different reaction buffer pHs (7.5 – 9.5, as indicated). (d) An alternative RNA substrate sequence was incubated with increasing amounts of *Tb*EndoV (0 – 2000 nM, as indicated). Reaction products were analyzed by 20% denaturing PAGE. Positions of the reaction substrate (S) and product (P) are shown.

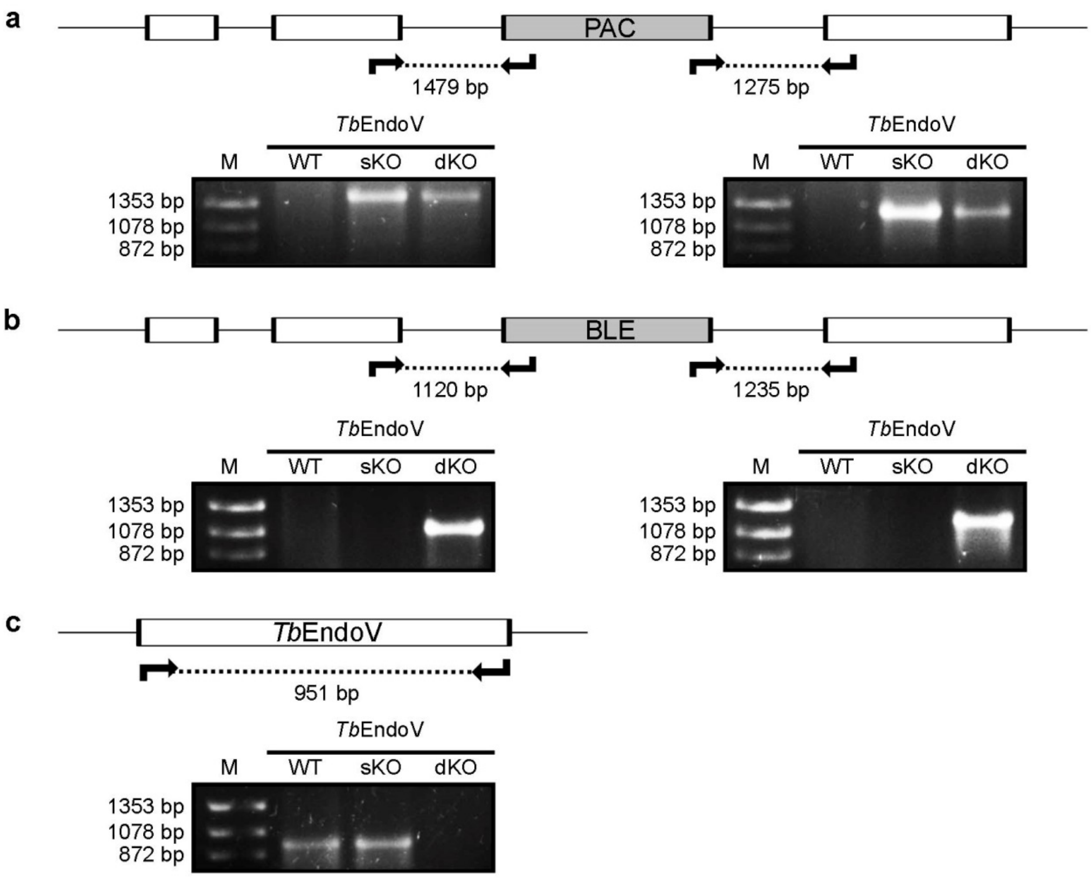

Supplementary Figure S3. Analysis by PCR of *Tb*EndoV knockout bloodstream cells. (a) Genomic DNA from single (sKO) and double-replacement (KO) clones where puromycin (PAC) has integrated at the right locus produces 1479 and 1275 bp PCR fragments with two different sets of primers. (b) PCR analysis of clones obtained after a second-round inactivation where the phleomycin (BLE) cassette has correctly recombined yields 1120 and 1235 bp amplification products. (c) Only the double knockout clone does not retain any copy of *Tb*EndoV as shown by the absence of PCR product (951 bp) after the PCR assay with internal *Tb*EndoV-specific primers.

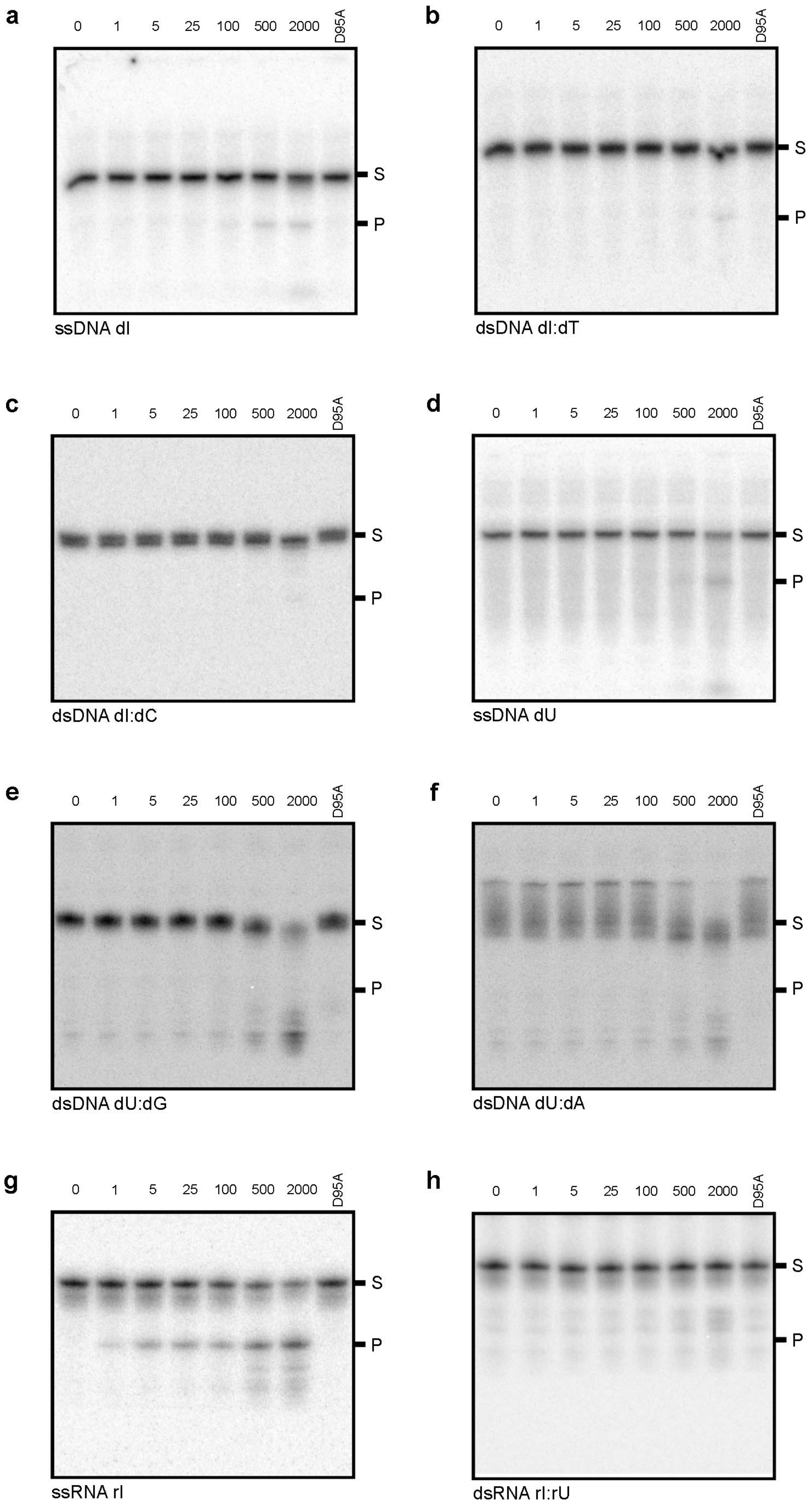
a
0 1 5 25 100 500 2000 D95A
S
P
ssDNA dI
b
0 1 5 25 100 500 2000 D95A
S
P
dsDNA dI:dT
c
0 1 5 25 100 500 2000 D95A
S
P
dsDNA dI:dC
d
0 1 5 25 100 500 2000 D95A
S
P
ssDNA dU
e
0 1 5 25 100 500 2000 D95A
S
P
dsDNA dU:dG
f
0 1 5 25 100 500 2000 D95A
S
P
dsDNA dU:dA
g
0 1 5 25 100 500 2000 D95A
S
P
ssRNA rI
h
0 1 5 25 100 500 2000 D95A
S
P
dsRNA rI:rU

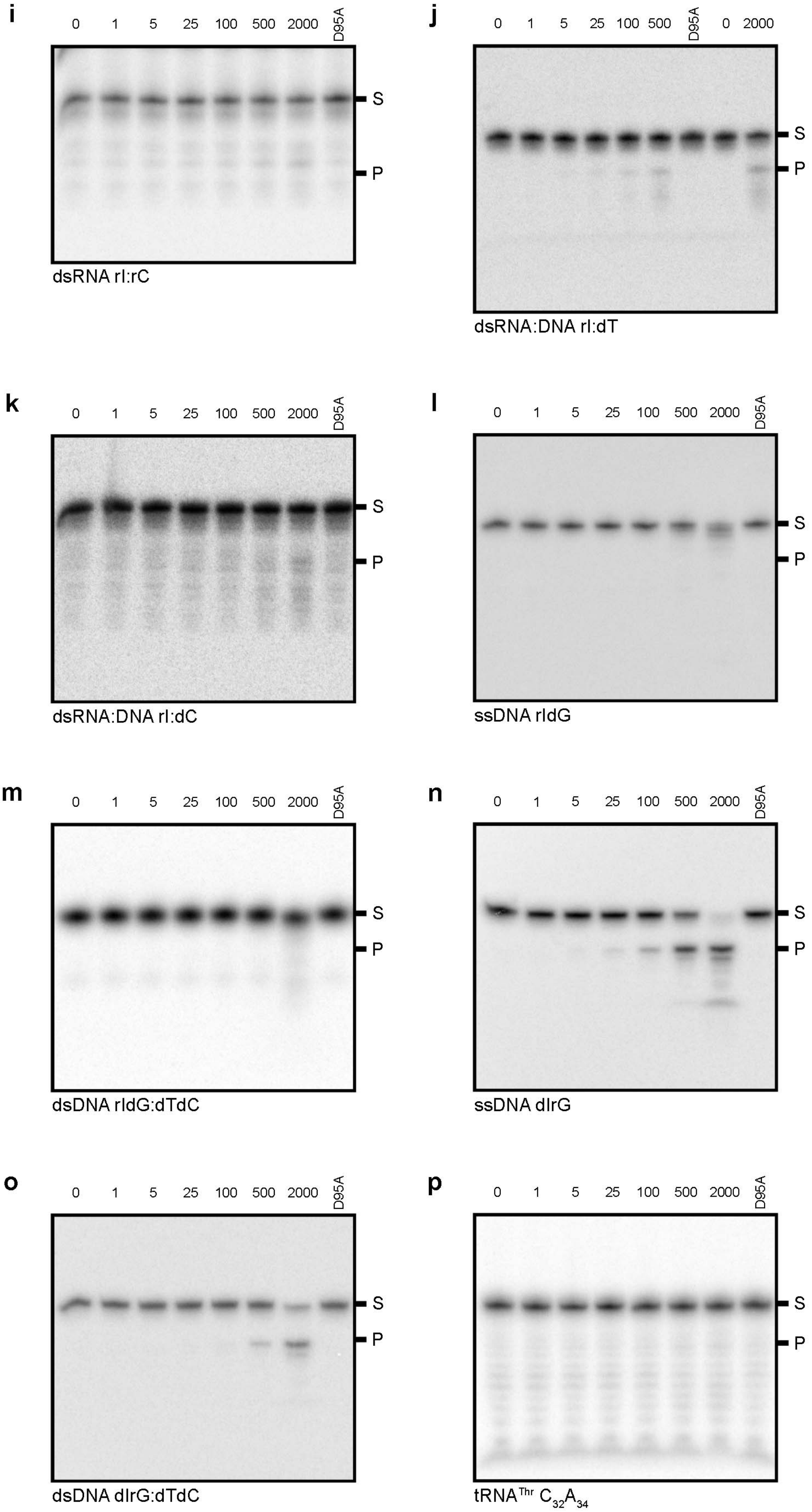
i
0 1 5 25 100 500 2000 D95A
S
P
dsRNA rI:rC
j
0 1 5 25 100 500 D95A 0 2000
S
P
dsRNA:DNA rI:dT
k
0 1 5 25 100 500 2000 D95A
S
P
dsRNA:DNA rI:dC
l
0 1 5 25 100 500 2000 D95A
S
P
ssDNA rIdG
m
0 1 5 25 100 500 2000 D95A
S
P
dsDNA rIdG:dTdC
n
0 1 5 25 100 500 2000 D95A
S
P
ssDNA dIrG
o
0 1 5 25 100 500 2000 D95A
S
P
dsDNA dIrG:dTdC
p
0 1 5 25 100 500 2000 D95A
S
P
tRNA^Thr C32A34

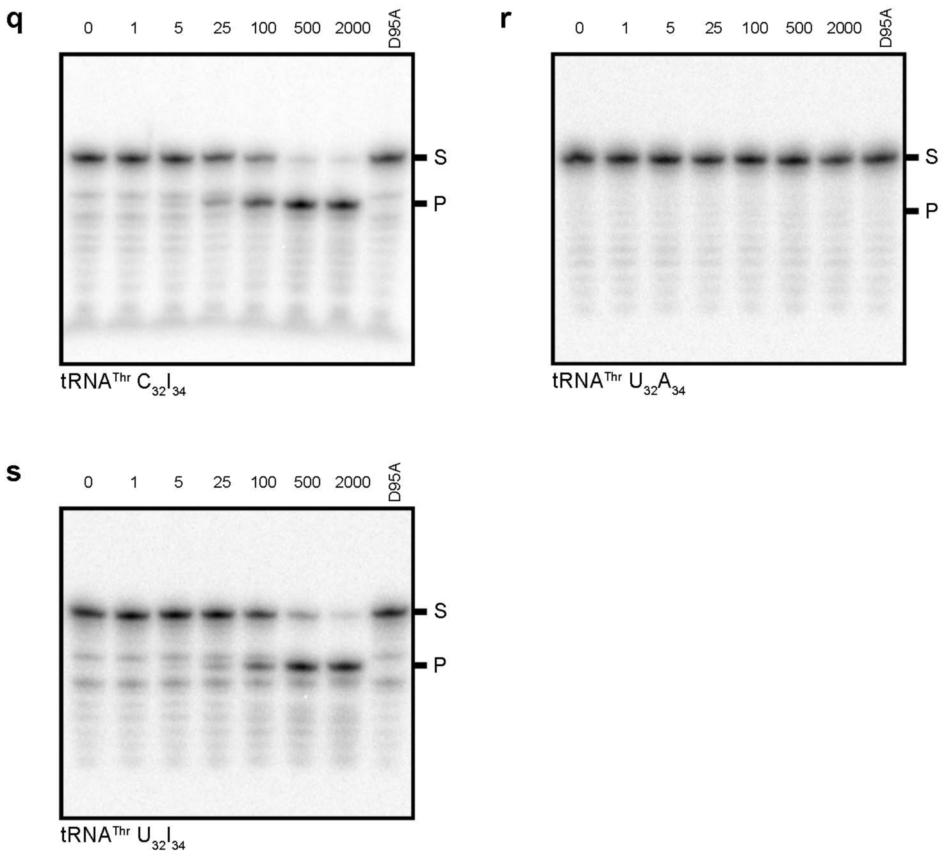

Supplementary Figure S4. Full size images of *Tb*EndoV activity gels presented in figures 2 (a-k), 3 (l-o) and 4 (p-s) of the manuscript.

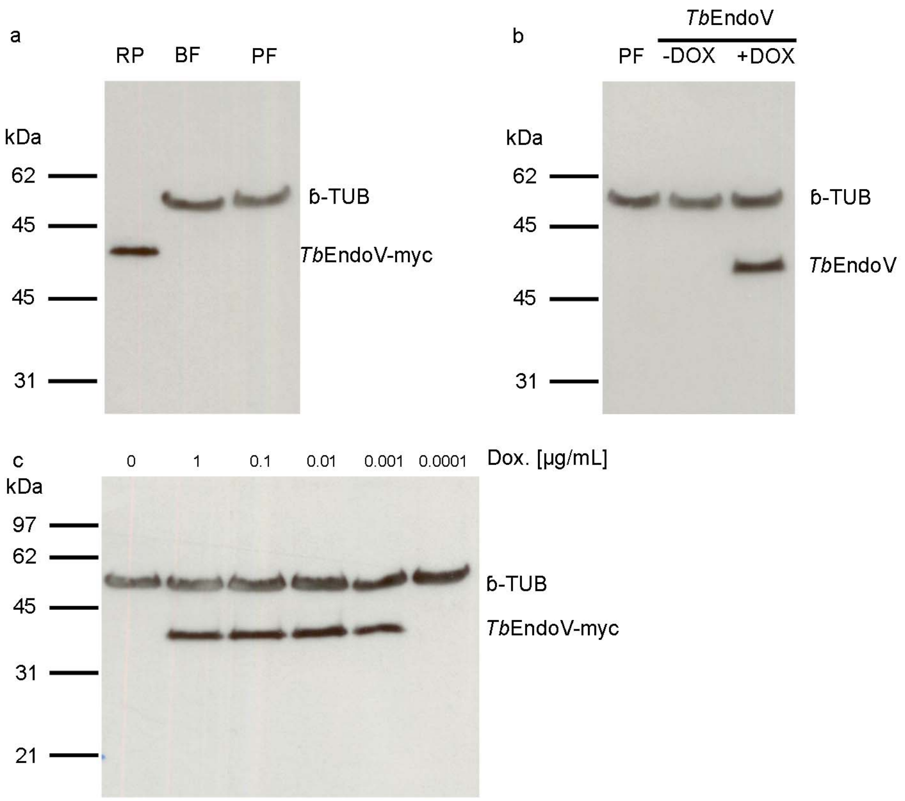

Supplementary Figure S5. Full size images of western blots presented in figure 5 of the manuscript. (a) Western blot of the expression levels of EndoV in bloodstream (BF) and procyclic (PF) forms using an anti-*Tb*EndoV polyclonal antibody generated in our laboratory. Recombinant purified *Tb*EndoV (20 ng) (RP) was included as positive control. (b) Expression of native *Tb*EndoV and (c) *Tb*EndoV-myc detected in doxycycline induced *Tb*EndoV- and *Tb*EndoV-myc-overexpressing PF cells using the anti-*Tb*EndoV polyclonal antibody or an anti-myc monoclonal antibody, respectively. Anti-β-tubulin was used as loading control in all western blotting analysis.
